# Computational modeling to predict the functions and impact of drug transporters

**DOI:** 10.1186/s40203-015-0012-3

**Published:** 2015-09-04

**Authors:** Pär Matsson, Christel A S Bergström

**Affiliations:** Department of Pharmacy, Uppsala University, Box 580, SE-751 23 Uppsala, Sweden; Uppsala University Drug Optimization and Pharmaceutical Profiling Platform (UDOPP) – a node of the Chemical Biology Consortium Sweden, Uppsala, Sweden

**Keywords:** Drug transport, Membrane transporter, Carrier-mediated transport, Structure-activity relationship, Ligand-based modeling, Structure-based modeling

## Abstract

Transport proteins are important mediators of cellular drug influx and efflux and play crucial roles in drug distribution, disposition and clearance. Drug-drug interactions have increasingly been found to occur at the transporter level and, hence, computational tools for studying drug-transporter interactions have gained in interest. In this short review, we present the most important transport proteins for drug influx and efflux. Computational tools for predicting and understanding the substrate and inhibitor interactions with these membrane-bound proteins are discussed. We have primarily focused on ligand-based and structure-based modeling, for which the state-of-the-art and future challenges are also discussed.

## Introduction

Transport proteins, which are expressed in all tissues of the body, facilitate the transmembrane transport of essential solutes such as nutrients and signal substances. They also play an important role in the removal of metabolites and toxicants from cells and tissues. Transporters of xenobiotics such as drug molecules are typically divided into influx and efflux transporters, where the former mediate the transport of compounds into the cell interior and the latter secrete compounds out from the cell. In addition, the role of transporters in the flux of compounds between subcellular organelles is increasingly recognized.

The main gene superfamilies involved in the transport of drugs and similar molecules are the ATP-binding Cassette (ABC) family and the solute carrier (SLC) family (Giacomini et al. 2010, Schlessinger et al. 2010, Hediger et al. 2013, Hillgren et al. 2013). Seven ABC subfamilies have been identified in humans, all of which are involved in the secretion of compounds from the cytosol, typically to the cell exterior. The most important ABC transporters for the efflux of drugs and drug-like molecules are P-glycoprotein (MDR1/P-gp; ABCB1), Breast Cancer Resistance Protein (BCRP; ABCG2), Bile Salt Export Pump (BSEP; ABCB11), and the members of the multidrug resistance-associated protein family (MRP; ABCC) (Giacomini et al. 2010, Hillgren et al. 2013).

About 40 human ABC transporters are known to date, while more than 350 SLC transporters have been identified (Schlessinger et al. 2010, Hediger et al. 2013, Schlessinger et al. 2013a, b). Only a small number of these have so far been proven to be involved in drug distribution, disposition and elimination. The SLCs have more diverse functions than the ABCs; the majority mediates cellular influx, while others are bidirectional or predominantly mediate cellular efflux. For drug-like molecules, the most important SLCs are encoded by genes in the subfamilies SLCO (predominantly negatively charged substrates), SLC15 (di- and tripeptides), SLC22 (mainly organic cations and anions) and SLC47 (mainly organic cations). The names and tissue expression patterns of transport proteins of demonstrated importance for drug transport and/or drug-drug interactions (DDI) are listed in Table 1.

**Table 1:** Nomenclature and protein-based tissue expression of common drug transport proteins

Gene name	Protein name	Organ	Expression level	Reference
ABCB1	MDR1^a^	Small intestine	Moderate	Oswald et al. 2013
		Liver	Low to moderate	Pedersen 2013
		Kidney	Low	Human Protein Atlas
		Brain	High	Shawahna et al. 2011
ABCB11	BSEP	Small intestine	Not detected	Human Protein Atlas
		Liver	High	Human Protein Atlas
		Kidney	Not detected	Human Protein Atlas
		Brain	Low	Human Protein Atlas
ABCC1	MRP1	Small intestine	Moderate	Human Protein Atlas
		Liver	Not detected	Human Protein Atlas
		Kidney	High	Human Protein Atlas
		Brain	Low	Human Protein Atlas
ABCC2	MRP2	Small intestine	Low to moderate	Oswald et al. 2013
		Liver	Moderate to high	Pedersen 2013
		Kidney	Moderate	Human Protein Atlas
		Brain	Moderate	Human Protein Atlas
ABCC3	MRP3	Small intestine	Moderate	Human Protein Atlas
		Liver	Not detected	Human Protein Atlas
		Kidney	Moderate	Human Protein Atlas
		Brain	Low	Human Protein Atlas
ABCC4	MRP4	Small intestine	Data not found	
		Liver	Data not found	
		Kidney	Data not found	
		Brain	Low to moderate	Shawahna et al. 2011
ABCC5	MRP5	Small intestine	Low	Human Protein Atlas
		Liver	Not detected	Human Protein Atlas
		Kidney	High	Human Protein Atlas
		Brain	Low	Human Protein Atlas
ABCG2	BCRP	Small intestine	Moderate	Oswald et al. 2013
		Liver	Low to moderate	Pedersen 2013
		Kidney	Low	Human Protein Atlas
		Brain	Low to high	Shawahna et al. 2011
SLC15A1	PEPT1	Small intestine	High	Oswald et al. 2013
		Liver	Moderate	Human Protein Atlas
		Kidney	Moderate	Human Protein Atlas
		Brain	Moderate	Human Protein Atlas
SLC22A1	OCT1	Small intestine	Moderate	Human Protein Atlas
		Liver	Moderate	Human Protein Atlas
		Kidney	Moderate	Human Protein Atlas
		Brain	Low	Human Protein Atlas
SLC22A2	OCT2	Small intestine	Not detected	Human Protein Atlas
		Liver	Not detected	Human Protein Atlas
		Kidney	High	Human Protein Atlas
		Brain	Low	Human Protein Atlas
SLC22A3	OCT3	Small intestine	Moderate	Human Protein Atlas
		Liver	Moderate	Human Protein Atlas
		Kidney	High	Human Protein Atlas
		Brain	Moderate	Human Protein Atlas
SLC22A8	OAT3	Small intestine	Not detected	Human Protein Atlas
		Liver	Not detected	Human Protein Atlas
		Kidney	Moderate	Human Protein Atlas
		Brain	Low	Human Protein Atlas
SLC47A1	MATE1	Small intestine	Moderate	Human Protein Atlas
		Liver	Low	Human Protein Atlas
		Kidney	High	Human Protein Atlas
		Brain	Low	Human Protein Atlas
SLC47A2	MATE2	Small intestine	Low	Human Protein Atlas
		Liver	Not detected	Human Protein Atlas
		Kidney	Low	Human Protein Atlas
		Brain	Moderate	Human Protein Atlas
SLCO1B1	OATP1B1	Small intestine	Not detected	Human Protein Atlas
		Liver	Moderate	Human Protein Atlas
		Kidney	Not detected	Human Protein Atlas
		Brain	Not detected	Human Protein Atlas
SLCO1B3	OATP1B3	Small intestine	Not detected	Human Protein Atlas
		Liver	High	Human Protein Atlas
		Kidney	Not detected	Human Protein Atlas
		Brain	Not detected	Human Protein Atlas
SLCO2B1	OATP2B1	Small intestine	Not detected	Human Protein Atlas
		Liver	Low	Human Protein Atlas
		Kidney	Not detected	Human Protein Atlas
		Brain	Moderate	Human Protein Atlas

Tissue expression data taken from The Human Protein Atlas (www.proteinatlas.org) accessed July 14, 2015. Tissue data from the protein atlas are based on antibody staining of normal human tissue and this source was used together with listed references based on proteomics from which data on transport proteins are emerging. Tissue expression is only shown for small intestine, liver, kidney and brain; the transport proteins may be expressed in other tissues as well. Not detected means that the protein has been analyzed but the level is too low to be detected with the used method. Data not found means that we were not able to find reported tissue expression data when this review was prepared. Conflicting results were reported for BCRP where The Human Protein Atlas showed low expression whereas significant amount of protein was observed by Shawahna et al. (2011)

^a^MDR1 is also known as Pgp

## Review

### Transporters in Drug Disposition

**Fig. 1:**
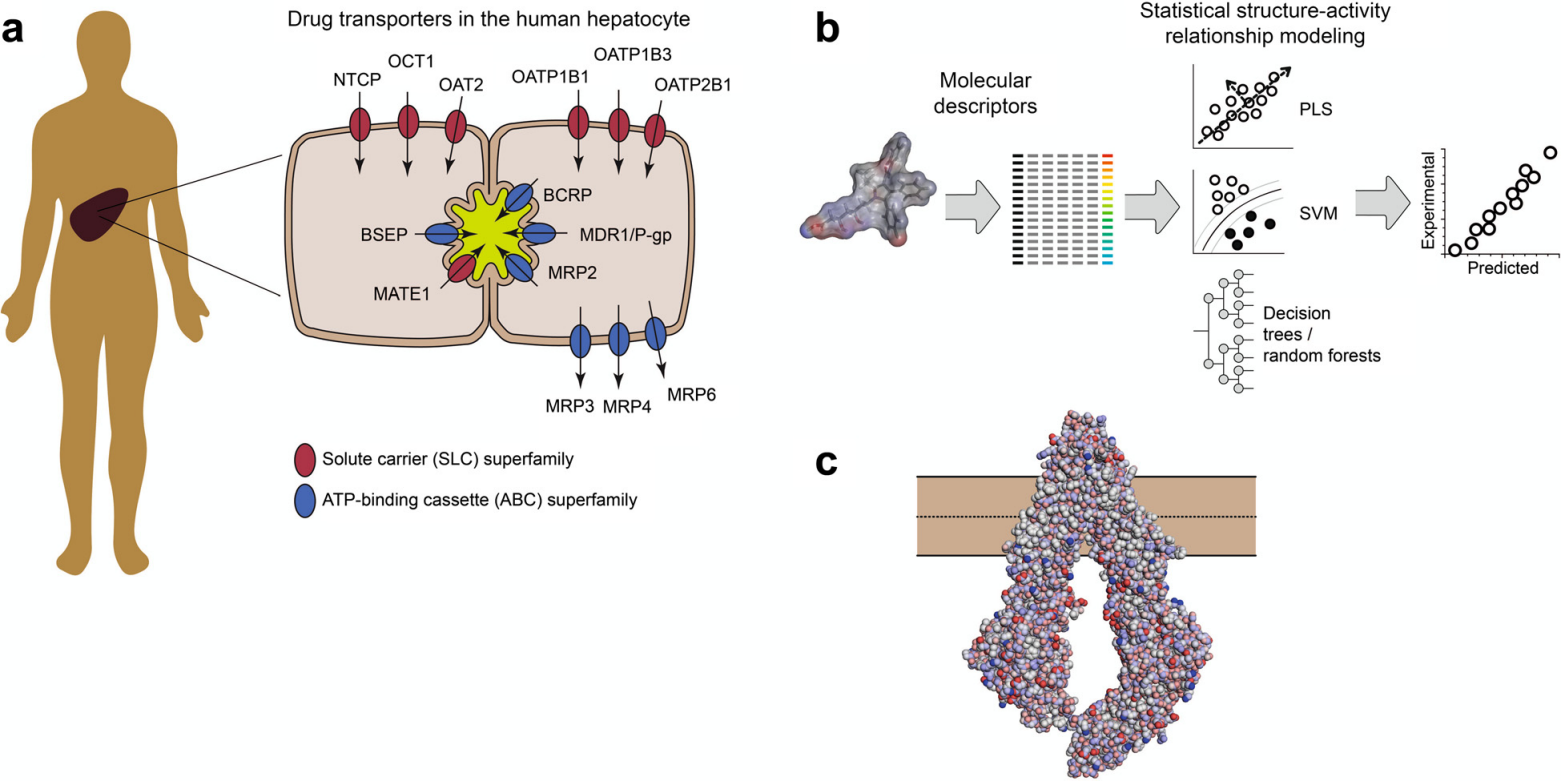
**a** Expression of transporters of importance for the handling of drug molecules, exemplified by the expression of such proteins in the liver. **b** Schematic procedure of ligand-based modeling of drug-transporter interactions. Molecular descriptors that encode fundamental molecular properties (*e.g.*, size, shape, polarity, charge) or structural features (*e.g.*, presence of specific substructures) are calculated for a set of drug molecules (ligands and non-ligands). Multivariate regression or classification methods are then used to relate the molecular descriptors to the measured activity (*e.g.*, affinity for the transporter, transport rates, or a binary classification: inhibitor/non-inhibitor or substrate/non-substrate). Commonly applied statistical methods include Partial Least Squares (PLS) projection, Support Vector Machines (SVM) and Decision Trees/Random Forests. Once properly validated (using, *e.g.*, cross-validation and external test set procedures), the models can be used to predict drug-transporter interactions for new molecules. **c** Protein structures of transporters (*e.g.*, mouse MDR1/P-gp, Protein Data Bank ID 4KSD) are used to predict ligand-transporter binding in computational docking experiments. When crystal structures are lacking, structures can be inferred from homologous proteins (homology/comparative modeling). The interactions between ligands and transporter binding sites are scored based on the complementarity of functionalities (*e.g.*, hydrogen bond formation, charge interactions and hydrophobic interactions) and the energies needed for the ligand to adopt a favorable conformation

The importance of transporters in drug absorption, disposition and elimination has been realized relatively recently, and a great deal of effort has been put into understanding their contribution to pharmacokinetics (PK), pharmacodynamics (PD) and drug-related toxicity over the last decade (Giacomini et al. 2010). Influx transporters can enable the permeation of compounds with low rates of diffusion across the lipoidal membrane (typically hydrophilic, polar, charged and/or large molecules), and may thus enable the oral absorption and tissue exposure of such molecules. These transporters have also been shown to cooperate with metabolic enzymes and hence they not only facilitate absorption from the gut but also enable the elimination of drug compounds in metabolically active tissues such as the liver (Benet 2009, Neve et al. 2013, Nordell et al. 2013, Li et al. 2014). Conversely, efflux transporters limit the intestinal absorption of substrate drugs, but also limit access to other tissues and are particularly involved in the limited distribution into the brain (Begley 2004, Hermann et al. 2006, Mahringer et al. 2011). They also contribute to the complex interplay between cellular influx, transcellular diffusion, drug metabolism and excretion of metabolites in pharmacokinetically important tissues such as the liver and kidneys (Masereeuw and Russel 2012, Pedersen 2013). A schematic overview of the expression pattern of ABC and SLC drug transporters expressed in the liver is shown in Fig. 1a.

The important, complex roles of transporters in the disposition of drug molecules in the body make it of great interest to study these processes in silico, with the ultimate goal of predicting PK profiles and possible DDIs even before the compound is synthesized. A wide variety of computational approaches has been applied for this purpose, as extensively reviewed in (Montanari and Ecker 2015). The toolbox available for molecular-level modeling of drug-transporter interactions is the same as that for other applications of structure-activity relationship (SAR) modeling, and is typically divided into ligand-based (Fig. 1b) and structure-based (Fig. 1c) approaches. Below, we discuss the respective applications, advantages and disadvantages of these approaches, along with some recent developments that should lead to improved models in the near future.

### Ligand-based modeling of drug-transporter interactions

As the name implies, ligand-based approaches use information about the structure and molecular properties of the ligands to explain their interactions with the transporters. Statistical methods are used to relate measurements of drug transport or drug-mediated transporter inhibition with numerical descriptions of the ligand chemical structures. The assumption is that such molecular descriptors will contain information that is relevant for explaining the drug-transporter interaction – *e.g.*, hydrogen bonds, charge and hydrophobic interactions, and steric effects. A wide variety of approaches are available for both the structural description and the statistical model development, and the experimental data used to train the models also come in several different shapes and flavors. Some advantages and disadvantages are, however, common to all ligand-based approaches. Explicit information about the transporter structure is not necessary, which is a clear advantage since, for the majority of the drug transporting proteins, relevant crystal structural information is still lacking. The models are typically of a multivariate nature. Methodologies commonly applied in ligand-based modeling include partial least squares projection to latent structures (PLS), support vector machines/regression (SVM/SVR), artificial neural networks (ANN) and random forests (RF). Importantly, statistical SARs like these are trained on a specific set of measured data and their applicability will be determined by the compounds included, the experimental method used, and the quality of the training data. For example, a model trained on a structurally related series of compounds will probably have limited predictivity outside that series, but should be able to identify series-specific details that the more general models could miss. In contrast, models trained on structurally diverse compound sets will better identify global trends and can be used, for example, to identify compound series that are likely to exhibit transporter liabilities. Conversely, the absence of protein-structure information entails that drug-transporter interactions cannot be calculated directly based on physical principles. Since molecular interactions are instead inferred from the properties and features of the ligands, models will be sensitive to the particular drug molecules used to train them.

Ligand-based modeling has been applied to most of the major ABC and SLC transporters implicated in drug transport (Giacomini et al. 2010, Hillgren et al. 2013, Sedykh et al. 2013). MDR1/P-gp in particular has been extensively studied in silico, see *e.g.* Gombar et al. 2004, Boccard et al. 2009, Matsson et al. 2009, Broccatelli et al. 2011, Broccatelli 2012. The reasons for this are two-fold: P-gp is one of the most important transporters in the cellular protection and detoxificafion process and, because it was the first efflux transporter to be identified, a large body of experimental data is available. More recently, as experimental data are becoming available, the same types of modeling approach have been applied to other drug-transporting ABCs and SLCs, including BCRP (Matsson et al. 2007, Matsson et al. 2009), MRP2 (Pedersen et al. 2008, Matsson et al. 2009) OCT1 (Ahlin et al. 2008), OCT2 (Suhre et al. 2005, Kido et al. 2011) OATs (Truong et al. 2008, Soars et al. 2014), OATPs (Karlgren et al. 2012, De Bruyn et al. 2013) and MATE1 (Wittwer et al. 2013).

Descriptions of chemical structure range from binary fingerprints that encode the presence or absence of certain substructural features in each transporter ligand, via descriptors of general molecular properties (including size, shape, lipophilicity, polarity and charge), to pharmacophores (describing the three-dimensional locations of ‘pharmacophore features’, *i.e.*, functionalities involved in charge interactions, hydrogen bonding or hydrophobic interactions) and molecular fields (describing the interaction potential of the ligand with ‘interaction probes’ placed in a grid around the molecule). The latter two approaches have the advantage of providing three-dimensional information about molecular interactions between the ligand and its environment (Dong et al. 2013). However, they strongly rely on accurate alignment of the transporter-interacting compounds to derive correct spatial information, and may thus be more suitable for series of structurally related ligands that bind the same site in the transporter than for modeling structurally diverse compounds that potentially bind to different regions of the transporting protein. In contrast, models based on general molecular descriptors may be advantageous for structurally diverse ligands that potentially interact with several different binding sites or with more diffuse ‘binding regions’ (Kido et al. 2011, Pedersen et al. 2013) as observed, for example, in the crystal structures of some ABC transporters (Aller et al. 2009).

Each way of representing the ligand structure has its advantages and disadvantages, and will thus be more or less suitable depending on the particular application and set of compounds to be modeled. For example, substructure fingerprints are sensitive to how frequently the different substructures occur in the sets of interacting and non-interacting compounds. If a substructure that is involved in ligand-transporter binding is rare in a set of interacting compounds, it may not be detected as statistically enriched. In contrast, substructural motifs that are common in a series of structurally related interacting compounds can be detected even when they are not directly involved in ligand-transporter binding (instead, they will be proxies for the particular compound series). Notably, consensus-based modeling approaches that combine different ways of representing ligand structures (*e.g.*, pharmacophore- and molecular descriptor-based models) have been shown to improve predictions of external validation sets (Broccatelli et al. 2011).

### Structure-based modeling of drug-transporter interactions

In contrast to the ligand-centric methods, structure-based modeling starts from spatial information about the protein structure of the transporter, most commonly derived using X-ray crystallography. This allows direct modeling of ligand-transporter interactions, for example through computational docking experiments in which ligands are introduced into the transporter structure and its binding pocket. The interactions are scored based on the complementarity between the ligand and the binding site with respect to size/shape, binding motifs and conformational strain.

Structure-based modeling thus has clear benefits in allowing direct inference of which ligand and target features are involved in an interaction. In contrast to ligand-centric modeling approaches, the scoring functions used are typically based on fundamental physics principles (concerning, *e.g.*, the energetics of inter-atomic interactions and conformational flexibility). Structure-based models are thus less sensitive to the choice of ligands than ligand-based models. Importantly, the results of a docking experiment strongly depend on the quality of the template structure. To date, most available transporter structures have been obtained from bacterial proteins that are distantly related to human drug transporters. However, recently, the structures of the mouse Mdr1/P-gp ortholog (Aller et al. 2009, Ward et al. 2013) and the human glucose transporter GLUT1 (SLC2A1) (Deng et al. 2014) have been revealed.

In the absence of human transporter structures, comparative (homology) modeling can be used; in this method, unknown protein structures are inferred based on their homology to crystallized template structures. The transporter sequence of interest is aligned to that of the template protein, and the unknown protein structure is modelled based on spatial constraints (obtained from the alignment to the template structure), atomic statistical potentials, and molecular mechanics (see, *e.g.*, Sánchez et al. 2000 and Schlessinger et al. 2013a, b for reviews). Such modelled structures should of course be used with some caution, especially if the aim is to derive ligand-transporter interaction information. Typically the overall protein fold is maintained at relatively low sequence homology (Schlessinger et al. 2010, Schlessinger et al. 2013a, b) but the template and model structures need to be closely related if atomic-scale resolution is to be maintained to allow high-quality modeling of ligand binding. Further, docking experiments are complicated in that most structures have been crystallized in the absence of prototypical substrates, and may thus reflect conformational states that are less relevant for substrate binding.

These caveats aside, structure-based modeling has been successfully used to identify new ligands for several transporters, including MDR1/P-gp (Dolghih et al. 2011, Ferreira, (Ferreira et al. 2013)) and the noradrenaline transporter NET/SLC6A2 (Schlessinger et al. 2011). It should be noted that, in the few cases where structure-based modeling has been used in conjunction with ligand-based approaches, predictivity statistics have been somewhat in favor of the latter (see *e.g.* Klepsch et al. 2014). However, the numbers indicate that docking-based predictions of transporter ligands are possible, and the spatial information inherent in the methodology provides an advantage over purely ligand-based methods for interpreting the predictions. Combination approaches using these complementary methodologies are thus likely to yield synergistic information (Tan et al. 2013, Klepsch et al. 2014) and as an example, ligand-based structure-activity relationship data have been used to prioritize structure-based predictions (Klepsch et al. 2014).

## Conclusions

Computational models of molecular-level interactions have been developed for a number of important transporters, using both ligand-based and structure-based methodologies. These models can be used to predict the likelihood of interactions between a new chemical entity and a particular transporter. Most of the datasets explored so far are based on transport inhibition measurements, where large numbers of compounds have been screened for their potential to inhibit the transport of a known substrate. Smaller datasets of substrate transport have also been modeled using similar methods, but currently these datasets are too small to allow general conclusions regarding the molecular determinants for influx or efflux. Methodological advances in different aspects of drug-transporter interaction modeling can be expected to continue to improve the quality of predictions as well as our understanding of the transport process at a molecular level. This includes improved description of ligand structures that will accurately capture the features involved in the ligand-protein interaction; improved scoring functions for molecular docking that will more precisely replicate ligand binding energies; and improved statistical techniques that will describe the nonlinear relationships between ligand features and drug binding and transport. Such technological advances will allow better use of the available drug-transporter interaction data.

However, the most noticeable improvements will come from extending the database of high-quality experimental data, *i.e.* from experimentally obtained descriptions of human transport protein structures (crystal structures of ABC and SLC transporters) and the interaction patterns of these transporters (increasing the size of the ligand datasets). Sufficiently large datasets of drug-mediated transporter inhibition are available for only a limited number of transporters. For the remaining transporter panel, modeling exercises are reliant on the merging of data from multiple sources – thus including cell type, assay type, and inter-laboratory variability in the training data. This is particularly true for the modeling of transported substrates, where large consistent datasets are as yet unavailable in the public domain. Structure-based modeling and ligand-docking approaches are limited by the availability of crystal structure information for human transporters (or for transporters closely enough related to provide atomic-level accuracy in homology models). Technological and methodological advances allowing structure determination for these membrane-bound proteins are central to the improvement of drug-transporter interaction predictions. Most importantly, such advances will facilitate an understanding of the molecular interactions taking place when drug compounds are transferred across cell membranes.

In summary, the wish-list of developments that would facilitate future modeling efforts includes: i) additional large and internally consistent datasets of ligand-transporter inhibition (ideally, such datasets should be characterized by inhibition mechanism to distinguish competitive inhibitors from inhibitors with possible allosteric or non-specific mechanisms); ii) large datasets of verified transported substrates; and iii) atomic resolution structures of relevant transporters, preferably captured in several states of the transport cycle, and with co-crystallized model ligands to provide experimental data to which binding poses predicted by virtual screens can be compared. Efforts to fulfill this wish-list are underway in several laboratories world-wide, and significant progress can thus be anticipated over the next few years.

## Abbreviations

ABC: ATP-binding cassette
ANN: Artificial neural network
BCRP: Breast cancer resistance protein
BSEP: Bile salt export pump
DDI: Drug-drug interaction
MRP: Multidrug resistance-associated protein
PLS: Partial least squares projection to latent structures
PD: Pharmacodynamics
P-gp: P-glycoprotein
PK: Pharmacokinetics
RF: Random forest
SAR: Structure-activity relationship
SLC: Solute carrier
SVM: Support vector machines
SVR: Support vector regression
